# Analysis of spatial variation of street landscape greening and influencing factors: an example from Fuzhou city, China

**DOI:** 10.1038/s41598-023-49308-6

**Published:** 2023-12-08

**Authors:** Bowen Jin, Jianwei Geng, Shan Ke, Hui Pan

**Affiliations:** 1 School of Economics and Managemen, Minjiang University, Fuzhou, 350108 China; 2 College of Landscape Architecture, Fujian Agriculture and Forestry University, Fuzhou, 350102 China

**Keywords:** Environmental impact, Urban ecology

## Abstract

Urban street greening is an important part of urban green infrastructure, and Green View Index (GVI) is widely used to assess urban street quality and ecosystem service value as an important indicator to quantify the perception of green street landscape from a pedestrian perspective. However, the distribution of street greenery is imbalanced. Therefore, to explore the differences in street greening levels within urban cities, we crawled streetscape data using the Internet to assess the spatial distribution patterns of urban street GVI using deep learning and spatial autocorrelation, and combined 11 surrounding environmental features with multi-source geographic data to further analyze the key factors influencing the spatial variation of block GVI using ordinary least squares, geographically weighted regression (GWR) models, and multi-scale geographically weighted regression (MGWR) models. The results show that the mean value of GVI in Fuzhou city is low (23.08%), with large differences among neighborhoods and a significant spatial autocorrelation. Among the regression models, MGWR has the best fit with an R^2^ of 0.702, where the variables of NDVI, house price, accessibility of water bodies and parks, and the proportion of built-up land have a greater impact on GVI, and the factors do not have the same spatial effect size. The results can provide a scientific basis for promoting green visual equity in different blocks.

## Introduction

Urban green infrastructure has been recognized as an important element in maintaining urban ecological security and ensuring sustainable urban development^1^, providing a variety of functions for urban ecosystem services, including not only ecological benefits such as climate regulation^2^, air purification^3^, noise reduction^4,5^, and urban heat island alleviation^6^, but also providing certain landscape aesthetics and social and recreational services^7,8^. As an important part of urban green infrastructure, urban road landscape plays an important role in enhancing the overall urban landscape, establishing urban image^9^, and improving public happiness^10^. However, with the rapid development of urbanization, unreasonable urban street planning has led to spatial differences in street green landscape^11^, which seriously affects the fairness of human perception of green landscape^12^. Therefore, accurate quantitative analysis of street green landscapes in different urban areas and investigation of the spatial differentiation characteristics of street green landscapes and their influencing factors play an important role in guiding urban street landscape planning and improving the high level of urban green space construction.

Since street green landscape can be directly perceived by residents, it is more applied by more scholars to evaluate street landscape based on questionnaires, such as scoring assignments^13,14^, but it is more influenced by individual subjective experience. Remote sensing, with its advantages of fast, real-time and large-scale monitoring, has been used to evaluate the quantity and morphology of street greenery in cities using leaf area index^15^, normalized vegetation index^16^, green space coverage^17^ and green space per person^18^, but these indicators ignore the vertical dimension of street greenery landscape layout. In contrast, the green view index (GVI), first proposed by the Japanese scholar Yoji Aoki^19^, collects image data by taking photographs in four directions at the line of sight level, extracts green pixels using Adobe Photoshop^20^, MATLAB^21^, and LiDAR^22^, and quantifies the percentage of green landscape from the pedestrian perspective, making it possible to quantify the green space of human perception possible. With the rapid development of Google, Baidu, and Tencent maps, images downloaded from Street View Big Data can provide great data support for analyzing GVI at the urban scale^23,24^.

However, many studies have shown that GVI is influenced by a combination of factors and its spatial distribution is not uniform^25,26^. Examples include socio-demographic variables^27^, economic level^28^, and building density and height^29^. In addition, the type of land use and site attributes of the city are also closely related to the potential factors of street green landscape^30^. For example, street-side green space, waterfront parks, etc. Secondly, relevant urban planners and landscape designers believe that the physical attributes of the street itself, such as width and grade attributes, also influence the suitability of road green landscape construction and explain the spatial variation of GVI better than factors such as socio-demographics^26^. On the other hand, due to the different development time of different urban areas, the different functional positioning of zoning will also affect the spatial differentiation of urban GVI. Related studies have mainly adopted correlation analysis^24^, multiple or stepwise regression^31^, and ordinary least squares (OLS)^32^. These methods are commonly applied to different regions with related influences, however, such correlations are assumed to be unchanged across spatial locations. In contrast, geographically weighted regression (GWR), a local regression model, captures the spatial relationships between the dependent and independent variables that vary when in different locations^33,34^. However the optimal bandwidth found by the GWR model is the same for each explanatory variable, while different explanatory variables have different scales of action. The multiscale geographically weighted regression model (MGWR) finds the optimal bandwidth for each explanatory variable, extending the GWR model spatially and providing new insights into the regression results^35^.

Based on this, this study takes the main urban area of Fuzhou city as the research object, crawls the street view data of Baidu Map through the Internet, extracts the GVI of each image using deep learning, analyzes its spatial distribution pattern using spatial autocorrelation, etc., and further explores the key factors affecting the spatial differentiation of green view rate within the city and its influence range using OLS model, GWR model and MGWR model. The results of the study can provide reference for optimizing the green landscape of roads in Fuzhou city.

## Materials and methods

### Study area

**Figure 1:**
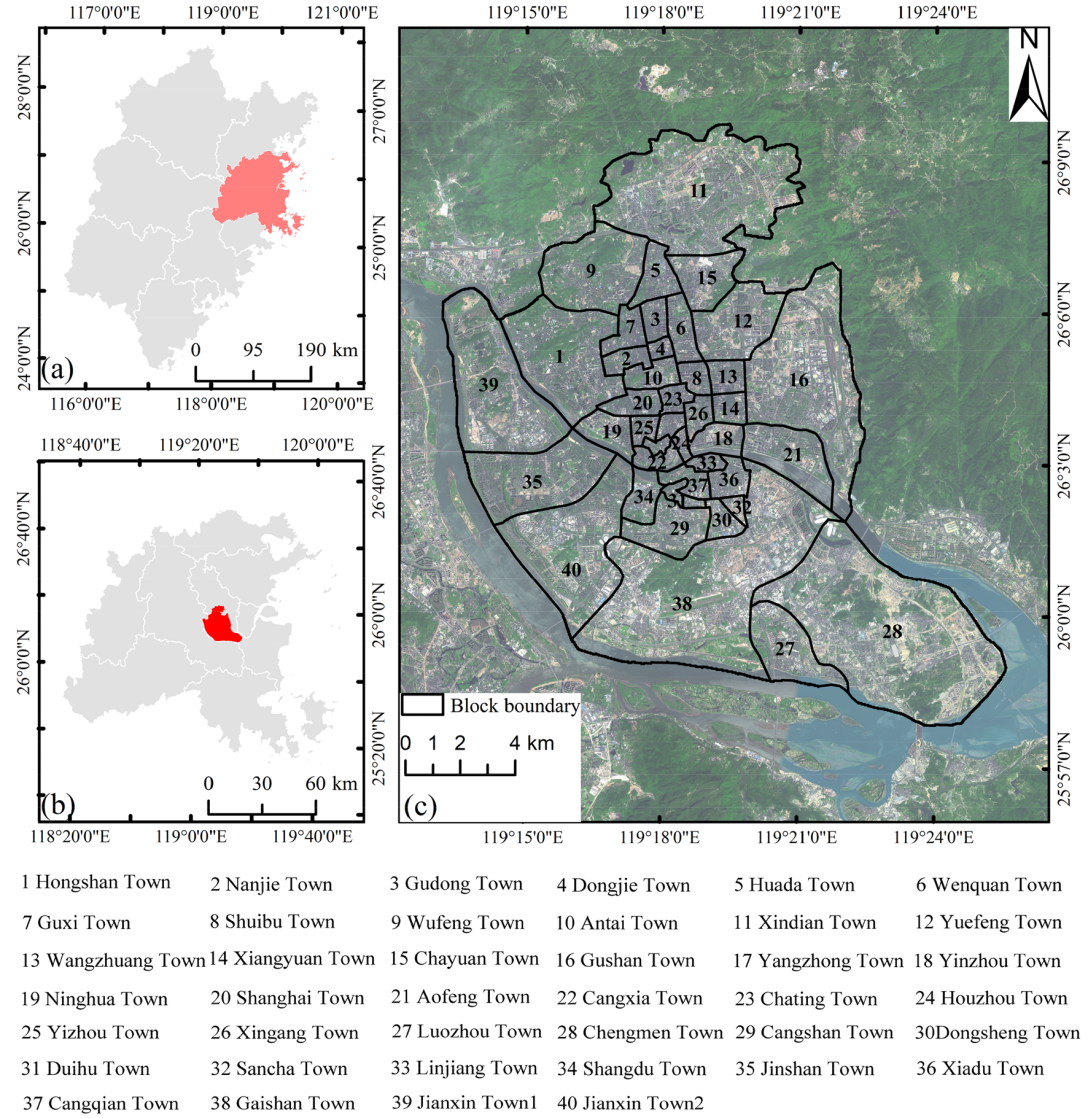
Location map of the study area; (**a**) Fujian Province; (**b**) Fuzhou city; (**c**) Block bound-ary.

Fuzhou city is located in the eastern part of Fujian Province, China, between 25° 15′-26° 39′ north latitude and 118° 08′–120° 31′ east longitude, in the downstream and coastal area of Min River. It has a subtropical monsoon climate with an average annual precipitation of about 900–2100 mm and an average annual temperature of 20–25 ℃. The street greenery is all evergreen trees, with no significant changes throughout the year. As a riverside coastal ecological garden city, we selected the economically developed and densely populated central city of Fuzhou as the study area. There are 40 blocks in the study area. (Fig. 1).

### Data sources and pre-processing

The road data were obtained from Open Street Map, checked by topology, and generated sampling points on the road with 100 m spacing, totaling 36,229 points. By extracting the coordinate information of the points and calling Baidu Street View API using python, 4 photos (0°, 90°, 180°, 270°) are obtained for each sample point. These photos are all taken by the camera installed on the top of the car, and the camera height is around 1600 mm, which is very close to the horizontal height of people's eyesight. Therefore, street view data was used instead of manual photography. In addition, we eliminated incomplete data such as tunnels, and finally collected a total of 144,916 street view data. The GDP and house price data were interpolated by the inverse distance weighting method to obtain a raster image with 30 m spatial resolution. The land use/cover data were obtained from Google Maps resource-sharing platform, and the 1 m high-precision images were obtained by supervised classification and visual interpretation. The accessibility of commercial, residential, and scenic areas is obtained by calculating the Euclidean distance of their points. Please see Table 1 for details.

**Table 1:** Data sources.

Data type	Format	Resolution	Source
Street View Pictures	.jpg	72ppi	https://lbsyun.baidu.com/products/panoramic
Block boundary, park, water and road	.shp		https://www.openstreetmap.org/ http://zygh.fuzhou.gov.cn/
POI (commercial, residential, scenic spots) GDP	.shp .tif	1000m	https://lbsyun.baidu.com/products/panoramic https://www.resdc.cn/
House price Land use/land cover NDVI	.shp .tif .tif	1m 30m	http://cc.anjuke.com/ Internal data of the subject group http://www.gscloud.cn/

### Methods

#### Extraction of GVI

**Figure 2:**
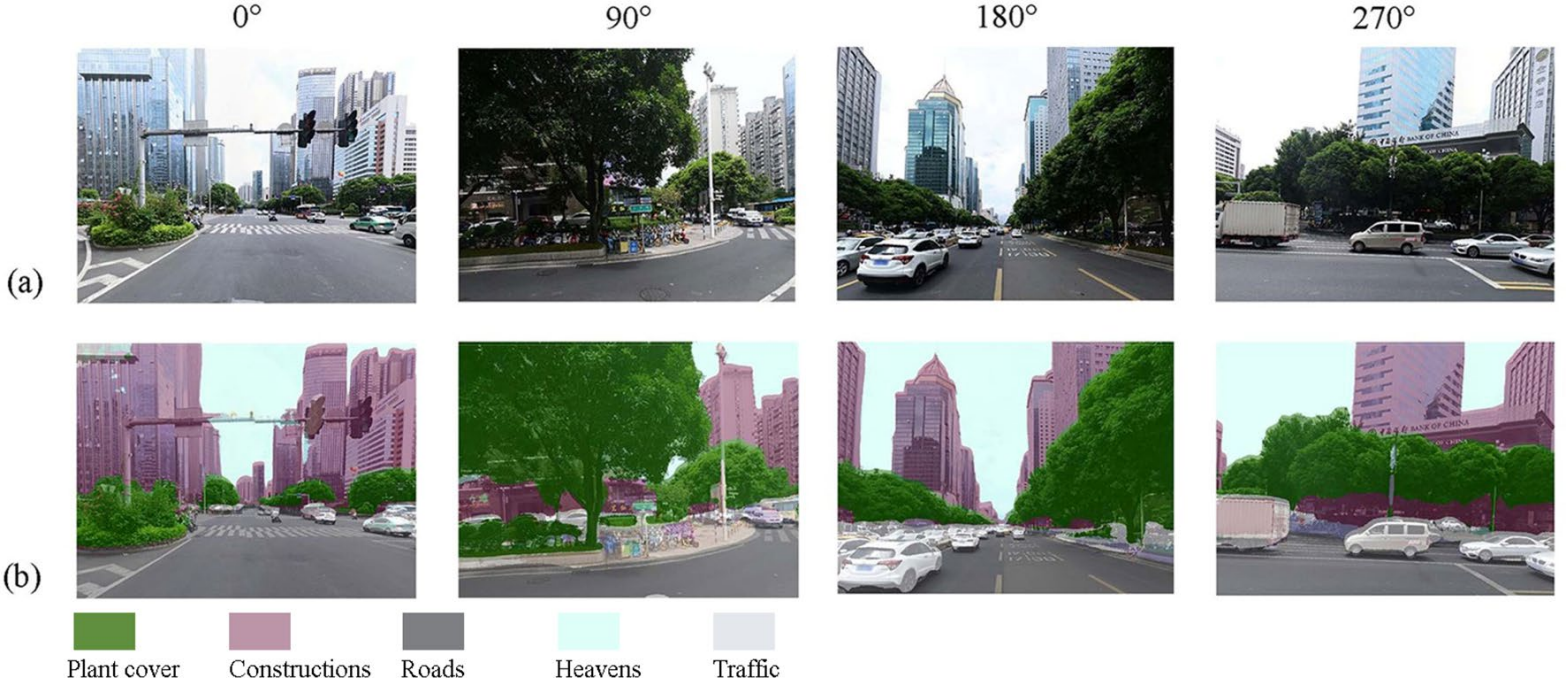
Semantic segmentation results; (**a**) Original image, (**b**) Segmented image.

The street green view is based on crawled Baidu Street View images and semantic image segmentation by full convolutional neural network FCN-8 s (Fig. 2). The FCN-8S network is trained based on the ADE-20 K dataset, which serves as a scene dataset that contains 150 target objects with a total number of 144,916 images, which is commonly used to carry out various aspects of scene perception, parsing, segmentation, multi-object recognition and semantic segmentation. As the FCN-8S network performs well in the Pascal visual object class, combining the semantic segmentation network with the street scene image can realize the prediction of semantic attributes of each pixel in the street scene image^31^.The method performs pixel-level classification of images and consists of multiple processing layers of semantic segmented images connecting the input and output layers to learn different levels of data information. Comparing the manual segmentation results with the semantic segmentation results FCN-8 s were found to be more accurate^36^. The amount of green for each sampling point is defined as the ratio of the number of plant pixels to the total number of pixels in the street image in the four directions (0°, 90°, 180°, 270°) of the sampling point, which is calculated as follows.

1 $$ Green \,View\, Index = \frac{{\mathop \sum \nolimits_{i = 1}^{4} Area_{g\_i} }}{{\mathop \sum \nolimits_{i = 1}^{4} Area_{t\_i} }} \times 100\% $$

In which, Green View Index is the result of green view extraction, ${Area}_{g\_i}$ is the number of green pixels in direction i among the four directions of a location point, and ${Area}_{t\_i}$ is the total sum of pixels in the photo taken in direction i.

#### Spatial autocorrelation model

Spatial autocorrelation is an important indicator to test whether an element is correlated with its neighboring spatial elements^33^. Spatial autocorrelation analysis of GVI can be used to describe the spatial homogeneity distribution of GVI in the study area. In this study, global spatial autocorrelation (Global Moran's I) and local spatial autocorrelation (Local Moran's I) were used to analyze the spatial correlation of GVI^37^.

Global Moran’s I is used to measure the interrelationship of spatial elements, and its value is between [−1, 1], the larger the absolute value, the stronger the spatial autocorrelation^38^.

2 $$I=\frac{n}{{S}_{0}}\frac{\sum_{i=1}^{n}{\sum }_{j=1}^{n}W\left(i,j\right)\left({X}_{i}-\overline{X }\right)\left({X}_{j}-\overline{X }\right)}{\sum_{i=1}^{n}{\left({X}_{i}-\overline{X }\right)}_{i}^{2}}$$

3 $${S}_{0}={\sum }_{i=1}^{n}{\sum }_{j=1}^{n}W\left(i,j\right)$$

where $n$ denotes the number of study objects, ${X}_{i}$ is the observed value, and $\overline{X }$ is the mean value of ${X}_{i}$. ${S}_{0}$ is the sum of all weights. $W\left(i,j\right)$ is the spatial connection matrix between study objects $i$, $j$.

The results of the Moran index were tested for significance with the following equation.

4 $$Z\left(I\right)=\frac{1-E\left(I\right)}{\sqrt{var\left(I\right)}}$$

where $E\left(I\right)=-1/\left(n-1\right)$; $var\left(I\right)$ is the variance of $I$. $\left| {Z\left( I \right)} \right|$ > 1.96 indicates significant spatial autocorrelation. When − 1.96 < $Z\left( I \right)$ < 1.96, it means the spatial autocorrelation is not significant.

Local Moran's I is the decomposition of Moran I into individual regional units. That is, LISA (Local Indicators of Spatial Association, LISA), LISA clustering map has five types of local spatial aggregation, which are high–high (HH), low–low (LL), low–high (LH), high-low (HL), and insignificant. For a certain spatial unit $i$.

5 $${I}_{i}=\frac{{X}_{i}-\overline{X}}{{S }_{3}}{\sum }_{j=1}^{n}W\left(i,j\right)\left({X}_{j}-\overline{X }\right)$$

6 $${S}_{3}=\frac{\left({\sum }_{j=1,j\ne i}^{n}{X}_{j}^{2}\right)}{\left(n-1\right)-\overline{{X }^{2}}}$$

where $n$, ${X}_{i}$, $\overline{X }$, $W\left(i,j\right)$ have the same meaning as Eqs. 5 and 6.

#### Selection of variables

Complex and diverse environmental variables and policies influence the changes of GVI. In this study, the GVI index is used as the dependent variable, and representative road width, Normalized Difference Vegetation Index (NDVI)^39^, the proportion of green space, the proportion of built space, commercial land accessibility, residential area accessibility, scenic spot accessibility, park accessibility, water accessibility^30^, house price^33^, and GDP are selected as independent variables^28^. Among them, the grade of road determines the size and scale of street green landscape space, NDVI represents the difference between real remote sensing image and actual perception, and the ratio of green space to built space represents the composition of landscape in the sampling grid in two-dimensional space. The indicators of commercial accessibility, residential accessibility, and scenic accessibility can reflect the role of land use nature on GVI. The GDP and housing price can reflect the economic development level of different regions. As an important ecological land in the city, the distance from the “blue-green” space also affects the change of GVI to a certain extent.

#### GWR model

Geographically weighted regression model (GWR) is a modified OLS model and is a local spatial analysis method. The common method of analyzing the relationship between two or more variables allows to explore the heterogeneity of spatial relationships by directly simulating and estimating locally non-stationary data, and is able to explore the heterogeneity of spatial relationships more highly^40,41^. Its formula is as follows.

7 $${Y}_{i}={\beta }_{0}\left({u}_{i},{v}_{i}\right)+{\sum }_{k}{\beta }_{k}\left({u}_{i},{v}_{i}\right){X}_{ik}+{\varepsilon }_{i}$$

where ${Y}_{i}$ is the dependent variable; ${X}_{ik}$ is the $k$ independent variables; $\left({u}_{i},{v}_{i}\right)$ is the geographical coordinates of the ith point; ${\beta }_{0}\left({u}_{i},{v}_{i}\right)$ is the intercept of the $i$th point; ${\beta }_{k}\left({u}_{i},{v}_{i}\right)$ is the coefficient of ${X}_{ik}$; ${\varepsilon }_{i}$ is the residual of the $i$th point.

By using weighted least squares, a regression equation was developed for each point considering only nearby observations. By various methods, each nearby observation is weighted by a distance function from the regression point. Common spatial weighting or distance decay methods include fixed Gaussian and adaptive bisquared kernel functions. The fixed Gaussian function can be written as.

8 $${W}_{ij}={\text{exp}}\left(-{\left(\frac{{d}_{ij}}{b}\right)}^{2}\right)$$

where ${W}_{ij}$ is the weight value of observation $j$ for estimating observation coefficient $i$. ${d}_{ij}$ is the distance between $i$ and $j$, and $b$ is the kernel bandwidth.

#### MGWR model

The traditional GWR model suffers from a bias in the analysis of spatial heterogeneity, which assumes that all influencing elements are constant at the spatial scale. Our study incorporates a multiscale geo-weighted regression model (MGWR) to address this issue by using different bandwidths rather than constant bandwidths, which can better explain the influence of the independent variable coefficients in space^35^. We used the MGWR 2.2 software to perform the calculations. The formula is as follows.

9 $${Y}_{i}={\beta }_{0}\left({u}_{i},{v}_{i}\right)+\sum_{k=1}^{m}{\beta }_{bwk}\left({u}_{i},{v}_{i}\right){X}_{ik}+{\varepsilon }_{i}$$

where $\left({u}_{i},{v}_{i}\right)$ is the geographic coordinate of the $i$th point; ${\beta }_{bwk}$ denotes the regression coefficient of the explanatory variable $k$; $m$ is the number of sampling points; $bwk$ is the bandwidth, and ${\varepsilon }_{i}$ is the residual of the $i$th point.

## Results

### Spatial distribution pattern of GVI

**Figure 3:**
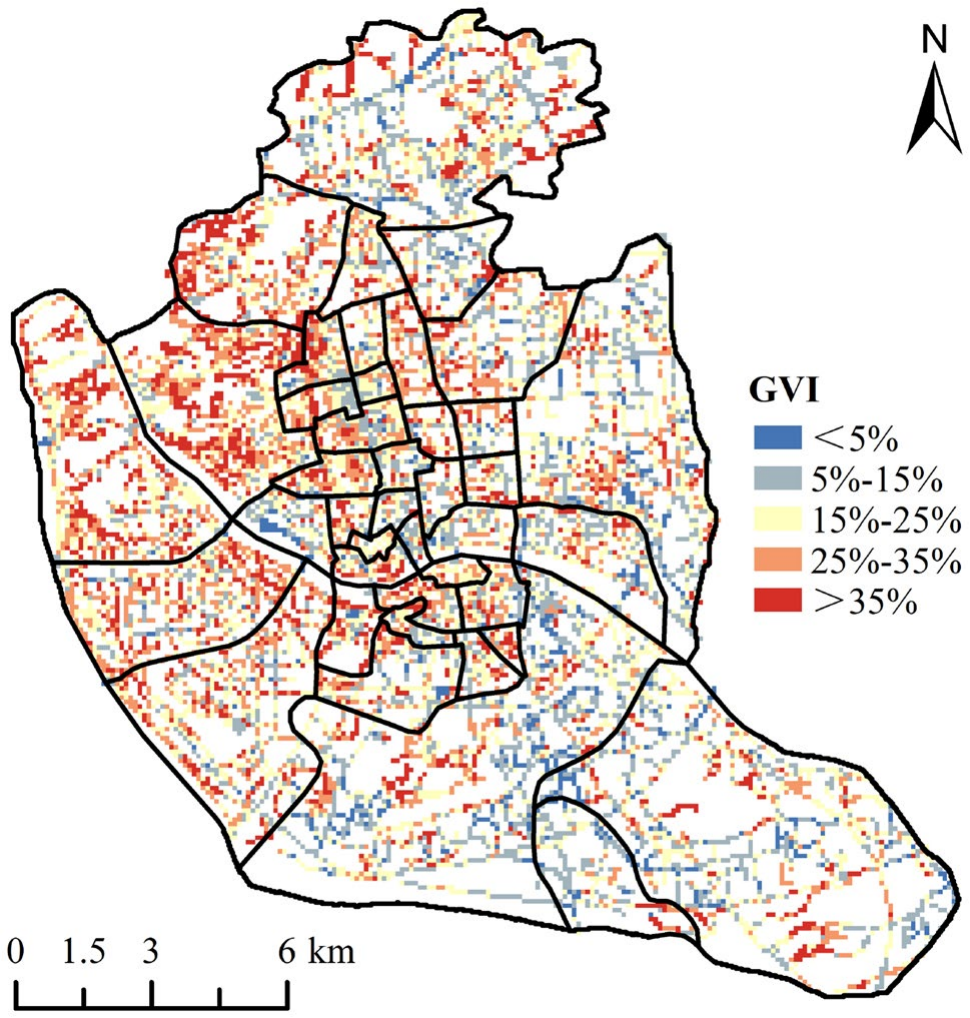
Spatial distribution of GVI in Fuzhou.

**Figure 4:**
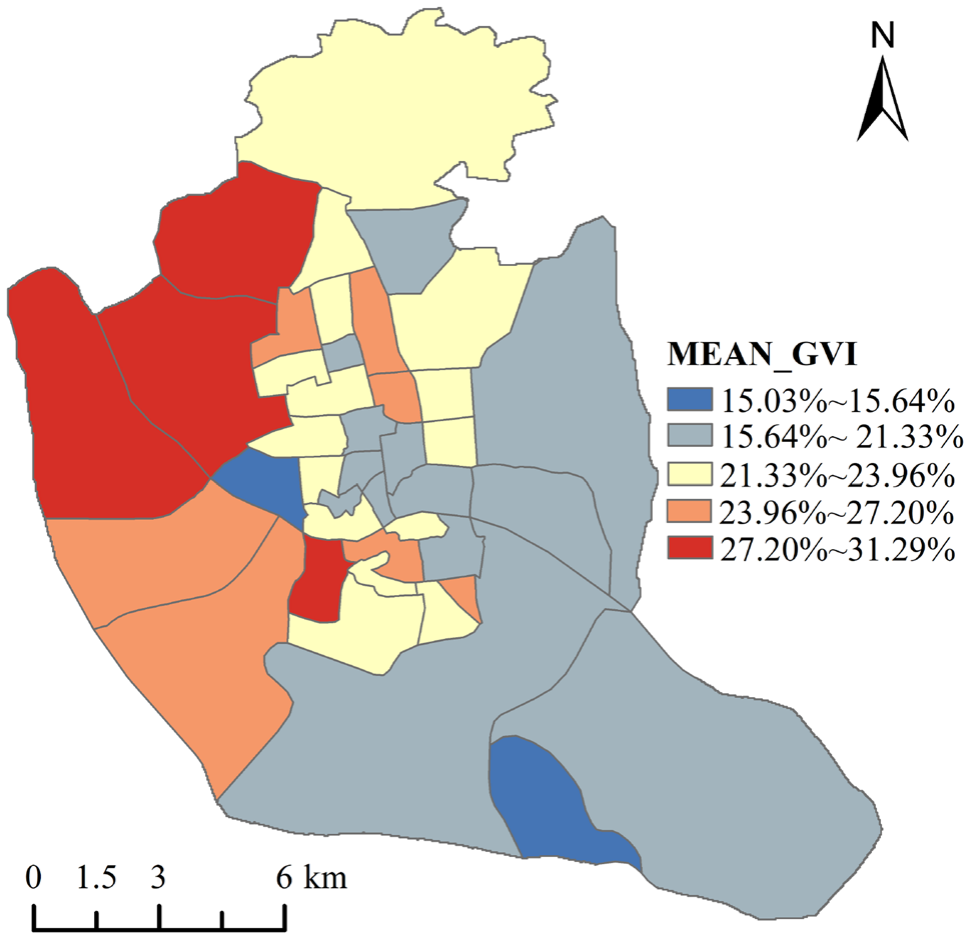
Average value of GVI by block in Fuzhou.

Referring to the grading approach in the relevant literature^31^, we classified the GVI into five levels. In our study area, GVI ranged from 0.08 to 94.63%, with a slightly lower mean value of 23.08%. Overall, the GVI was not uniformly distributed (Fig. 3). Using the zonal statistics tool, we counted the mean GVI values to each administrative division (Fig. 4). The highest mean GVI values were in Jianxin town (28.33%) and Shangdu street (28.53%) in Cangshan district and in Hongshan town (30.37%) and Wufeng street (31.29%) in Gulou district. the areas with the lowest mean GVI values were in Ninghua street (15.03%) in Taijiang district and in Luzhou Town in Changshan District (15.64%). It can be seen that although it is the same city, there are still large differences between the GVIs of different areas.

### GVI spatial autocorrelation

**Figure 5:**
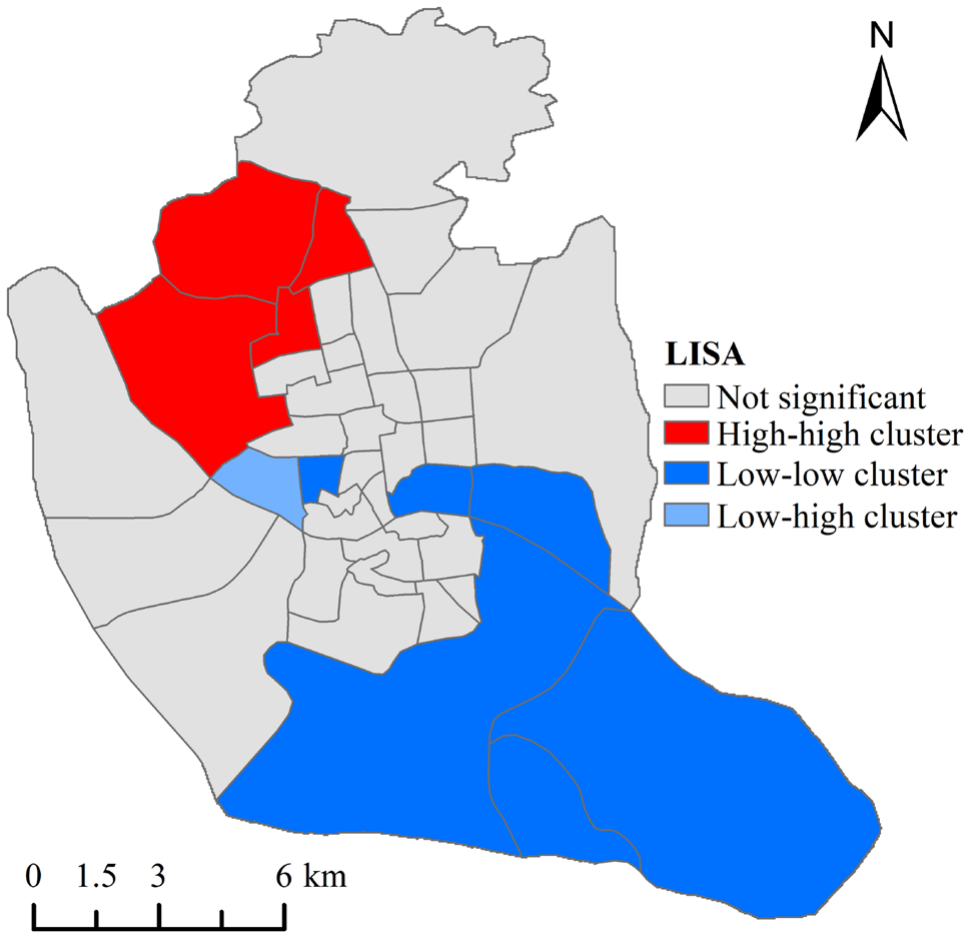
GVI clustering map.

We calculated the Moran index of the GVI of each block in the central city of Fuzhou through Geoda software, aiming to explore whether its spatial distribution has an aggregation effect. The results show that the Global Moran’s I is equal to 0.245 and the z-value is greater than 1.96, indicating that the GVI spatial autocorrelation of each block is significant. Secondly, we used the local spatial autocorrelation to draw the LISA clustering map of each block (Fig. 5). It is obvious that high-high clustering dominates in the northwestern part of the study area, and low-low clustering dominates in the southeastern part of the study area. The spatial autocorrelation of GVI in the intermediate areas is not significant, and we speculate that this phenomenon may be caused by the different development patterns of different areas.

### Analysis of factors influencing the spatial variation of GVI

#### OLS model

In order to explore in depth the key factors affecting the spatial differentiation of GVI, a total of 11 variables were selected for analysis from multiple perspectives, including the level of social development, regional economy, and accessibility to public resources. Table 2 shows the results of the OLS analysis between the GVI of each block and the selected influencing factors, and all factors passed the covariance test (VIF < 7.5) except for commercial accessibility (VIF = 7.991) and residential accessibility (VIF = 11.525). Among these variables, road width, NDVI, green space proportion, house price, and scenic accessibility have a positive relationship with GVI, with the most influential indicator being NDVI, with a standard coefficient of 0.708. followed by house price (0.426). The proportion of built space, GDP, park accessibility, and water accessibility are negatively correlated with GVI, with water accessibility having the greatest effect on GVI with a standard coefficient of -0.335.

**Table 2:** Linear regression analysis based on OLS.

Variables	Standard coefficient	VIF
Road width	0.27**	1.727
NDVI	0.708***	3.082
Green space proportion	0.22**	2.894
Building space proportion	− 0.215**	2.029
GDP	− 0.26*	2.085
House price	0.426**	3.06
Commercial accessibility	− 0.201	7.991
Residential accessibility	− 0.198	11.525
Scenic accessibility	0.268**	3.932
Park accessibility	− 0.228**	4.42
Water accessibility	− 0.335**	1.969
R^2^	0.696	
Adjust R^2^	0.577	
AICc	102.164	

***, **, and * represent 1%, 5%, and 10% significance levels, respectively.

#### GWR model and MGWR model

Due to OLS model shortcomings, the spatial relationships between explanatory variables could not be effectively tested. Therefore, we conducted a follow-up analysis using a geographically weighted regression model (GWR) and a multiscale geographically weighted regression model (MGWR). Based on the results of the OLS model in Sect.  3.2.1, we removed the variables with VIF values greater than 7.5 and performed GWR and MGWR for each variable with GVI. It can be seen that the fit of MGWR is better than the OLS and GWR models in both cases (Table 3).

**Table 3:** Fitting effects of different spatial regression models.

	OLS	GWR	MGWR
AICc	102.164	102.32	102.121
R^2^	0.690	0.692	0.702
Adjust R^2^	0.577	0.570	0.580

By comparing the regression models, we chose the MGWR model for the regression analysis of each variable, and we counted the coefficients of MGWR (Table 4). Table 4 shows that road width, NDVI, green space proportion, house price, and scenic accessibility have positive correlations with GVI, among which GVI has the highest correlation coefficients with NDVI and house price, which are 0.754 and 0.419, respectively, indicating that the growth condition of vegetation and housing price of the area are the main factors affecting the spatial variation of GVI. And the proportion of built space, GDP, park accessibility, and water accessibility have negative correlations with GVI. Among these indicators, the highest correlation coefficient is water accessibility, followed by park accessibility. This also means that the closer the distance to water bodies and parks, the higher the GVI.

**Table 4:** Summary statistics of MGWR model parameter estimation.

Variable	Mean	STD	Min	Median	Max
Road width	0.007	0.001	0.004	0.007	0.012
NDVI	0.754	0.003	0.748	0.754	0.760
Green space proportion	0.075	0.002	0.071	0.075	0.079
Building space proportion	− 0.224	0.002	− 0.227	− 0.224	− 0.220
GDP	− 0.188	0.001	− 0.192	− 0.188	− 0.185
House price	0.419	0.001	0.416	0.419	0.423
Scenic accessibility	0.212	0.001	0.210	0.212	0.215
Park accessibility	− 0.229	0.002	− 0.233	− 0.229	− 0.224
Water accessibility	− 0.359	0.000	− 0.360	− 0.359	− 0.358

#### Coefficient distribution of each variable in space

**Figure 6:**
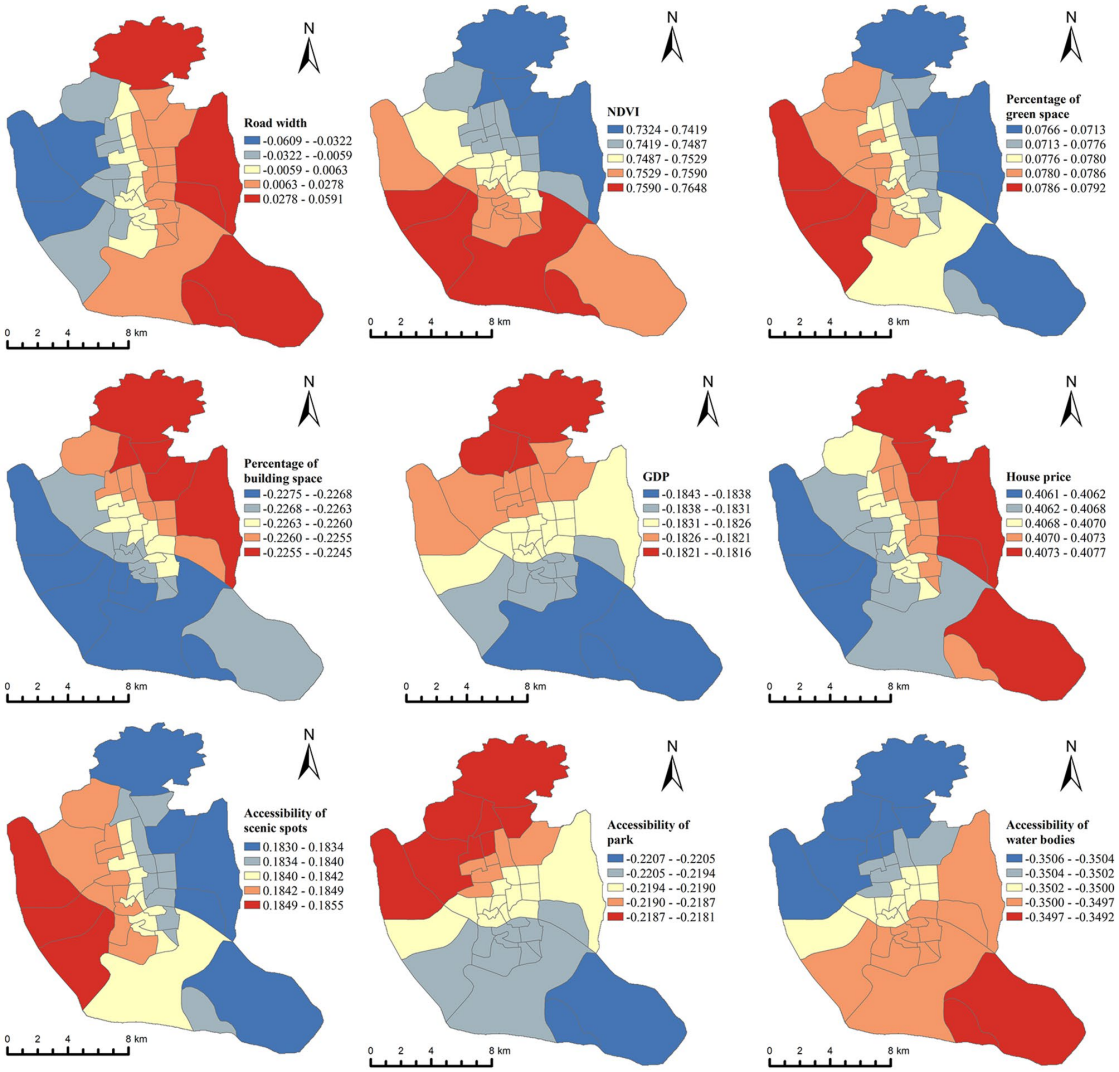
Regression coefficients of each variable in space.

To explore the effect and extent of each variable in space, we used the MGWR2.2 tool to visualize the effect and extent of each factor in space, using GVI as the dependent variable (Fig. 6).

As we can see through Fig. 6, the effect of the variables is not consistent across neighborhoods. For example, road width, in the western part of the study area, shows a negative inhibitory effect on GVI. That is, the smaller the road width in these areas, the higher the GVI. While in the eastern, northern and southern parts of the study area showed a positive promoting effect. It means that the wider the road, the higher the GVI. We believe that this phenomenon is caused by the timing of the construction of different neighborhoods. For example, the eastern and southern neighborhoods were built in a shorter period of time, and to avoid traffic problems similar to those in the old city, the roads were generally built with a wider width than the western neighborhoods. As a result, a larger view was obtained, and thus road width showed a positive correlation with GVI in the eastern and southern neighborhoods. In contrast, in the western neighborhoods of the study area, which were built longer, although the average width of the roads was narrower, the vegetation around the neighborhoods was better planted and grew for a longer period of time, and thus was more easily perceived.

NDVI is used as an important parameter to characterize the plant growth status, and the green space proportion reflects the greening level of different blocks. These two variables are often used to evaluate the quality of urban green spaces. In Fig. 6, we can see that NDVI has a positive contribution to GVI in general, with the most obvious contribution being in the south-central part of the study area; while the more obvious contribution of green space percentage to GVI is mainly in the western part of the study area. The proportion of built space is negatively correlated with GVI, and has a negative inhibitory effect on GVI in general. The areas with the most significant negative inhibitory effect are the northern and eastern parts of the study area. It means that the expansion of construction land in these areas will directly affect the decline of GVI. Therefore, from the perspective of green landscape visual equity, we suggest that the expansion of construction land scale in these areas should be reduced.

GDP and house price factors, as important indicators reflecting regional economic level, also influence the spatial divergence of GVI to some extent. Overall, the GDP factor has a negative inhibitory effect with GVI, while the house price has a positive promoting effect with GVI. This indicates from the side that regional economic development is accompanied by low investment in vertical street greening. In contrast, real estate developers invest a lot of money to enhance the GVI of the living environment in order to increase residents' desire to buy houses. Where the negative inhibitory effect of GDP on GVI is most evident in the northern and central regions of the study area. Therefore, we suggest that more attention should be paid to vertical greening of streets in these areas. The positive promoting effect of the house price factor on GVI is mainly located in the eastern part of the study area, mainly because the residential areas in the central and western regions are already saturated and developed at an earlier period, and the construction process does not focus on GVI, while the eastern regions are developed at a later period, and with the improvement of people's living standards, people are more and more willing to spend on greening, especially private green space construction, etc.

There is a positive correlation between the accessibility of scenic spots and GVI, implying that the closer the distance to the scenic spot, the lower the GVI. It indicates that the current planning in scenic areas not only does not harmonize with the surrounding environment, but also causes the decrease of surrounding GVI. Among them, the western part of the study area has the strongest impact effect. The reason for this phenomenon, we believe, is that the western region has a higher concentration of attractions and fewer available land resources. And there is a negative correlation between park accessibility and GVI, implying that the closer the distance to the park, the higher the GVI. This also indicates a higher level of greening on the periphery of the parks in Fuzhou. Among them, its impact effect is the highest in the northwestern part of the study area. In the southeastern part of the study area, the effect is smaller. The main reason for this phenomenon is the uneven distribution of parks in Fuzhou. More parks and green spaces are likely to exist in the Northwest, which may receive more attention and investment in urban planning and land use. This results in higher park accessibility and higher GVI. The accessibility of water bodies is also negatively correlated with GVI. It shows that the closer the distance to the water body, the higher the GVI. The area with the most significant effect is the southeastern area. The main reason is that the southeastern area is the confluence of Min River and Wulong River, so the accessibility of the water body is better. And the area with lower impact effect is mainly concentrated in the northwest of the study area.

## Discussion

### Comparison of GVI studies.

In our work, the GVI ranges from 0.08 to 94.63%, with a mean of 23.08%, slightly higher than Beijing, China (15.7%)^42^, Singapore (21.0%)^43^, and the Pearl River Delta region of China (11.3%)^28^; slightly lower than Hangzhou, China (28.2%)^31^, Hartford, Connecticut, USA (24.4%)^23^, Berkeley, California, USA (24.8%)^20^, and the average of major Chinese cities (27.6%)^21^. Meanwhile, Hangzhou, Pearl River Delta and Fuzhou are the major cities and regions in southern China, which are affected by climate and seasons and have less deciduous vegetation, while Beijing is located in the northern part of China and has more deciduous trees, which makes the GVI image data vulnerable to seasonal influences and hence differences. Moreover, Beijing and the Pearl River Delta are the most economically developed regions in China, while Fuzhou and Hangzhou are economically “backward” compared to the former. Therefore, we believe that economically developed areas have less space for increasing green space, and the population of Beijing and PRD is more dense than that of Fuzhou and Hangzhou, and their roads mainly serve the function of commuting by motor vehicles, so the original green landscape space is compressed.

### Relationship among GVI, NDVI, and green space proportion.

**Figure 7:**
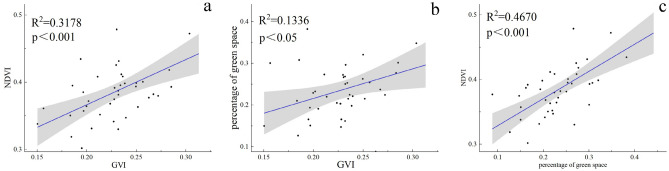
Two-by-two linear regression of GVI, (**a**), NDVI, (**b**), and green space share, (**c**).

In previous studies, NDVI and green space proportion are commonly used to describe the greening level of an area^44^. However, through our study (Fig. 7), we found that the relationship between GVI and NDVI and green space proportion is relatively weak, while the relationship between NDVI and green space proportion is strong, which indicates that GVI and NDVI and green space percentage indicate different perspectives of urban greening levels. This result is consistent with the results of related studies^42^. The correlation coefficient between its GVI and NDVI was 0.609. We believe that multiple factors contribute to the weak relationship between GVI and NDVI, and the percentage of green space. Differences in the specific location of the forest canopy layer where the human eye is located can cause such differences. For example, when we are under the forest canopy layer, it may be difficult to notice the green vegetation above the head from the human eye's perspective (flat view), but it can be clearly captured in remote sensing. Secondly, when we judge the surrounding GVI with a flat view, we often include the shrubs and grasses under the forest in the green pixels, however, both NDVI and green spatial occupancy are acquired based on two-dimensional remote sensing images, and cannot directly penetrate the forest canopy layer for identification. Therefore, under the influence of these factors, the relationship between GVI and NDVI and green spatial occupancy ratio is weakened.

### Analysis of GVI influence factors

Our results indicate that the spatial variation of GVI is related to many factors. Among them, GVI was positively correlated with the NDVI of the neighborhood, which is consistent with the findings of related studies^42^. Second, our study also finds that in socioeconomic terms, house prices contribute more to GVI than to GDP. This is not quite the same as the findings of related studies^28^. Local governments are important contributors to urban greening in China, and therefore, the regional economic level will directly affect the quality of street greening. However, economically developed regions tend to have a higher intensity of land use, while the economic development of Fuzhou city still has a large gap compared to the Pearl River Delta, and the intensity of urban development is much less than that of the Pearl River Delta^28^. Furthermore, GDP is not equivalent to the level of investment in green streetscape, so the explanatory power of GDP is low in our study. The reason for the high correlation of the house price variable is also considered to be due to the current development status of Fuzhou city. For example, the housing prices in cities such as the Pearl River Delta and Beijing are certainly high, but they were built earlier and, to the best of our knowledge, people did not require high greenery quality in residential areas in the early days, but more for commuting needs. With the development of social economy, people's willingness to spend more and more to improve the quality of greenery has become stronger and stronger, leading to the greenery quality in recent years has become one of the important indicators affecting the price of housing, similarly, the price of housing will also affect the quality of greenery around.

As a coastal and riverine garden city in southeast China, Fuzhou city has parks and water bodies that provide great ecological and social values to the city. In our study, we found that GVI, as an important indicator for evaluating green volume from a human-centered perspective, shows a negative correlation with the blue-green space of the city, implying that the closer the distance to urban parks and water bodies, the higher the GVI is, which also reflects from the side that the governance of parks and water systems in Fuzhou is based on the concept of human-centered perspective.

### Policy guidance measures.

To enhance the equity of GVI, relevant policymakers can develop greening planning policies to ensure that all communities in the city have access to equitable greening resources. This can include determining the distribution and size of greening areas, as well as ensuring the public accessibility of greening areas. Second, focus on disadvantaged communities. Relevant policymakers can focus on low-income or marginalized communities in greening planning. These communities often face insufficient greening resources and can be ensured equitable access to green space by increasing greening investments and providing additional support. In addition, policy makers can develop equitable distribution policies to ensure that greening resources are distributed fairly and equitably across the city. Ensure fair distribution of resources and avoid over-concentration of resources in certain areas. And, provide financial and technical support to help communities carry out greening projects. Finally, encourage community residents to participate in greening construction, such as vertical greening, rooftop gardens, and street-side greening to promote the overall greening level of the city and enhance the equity of GVI.

### Limitations and prospects

There are several limitations in our study. First, the street view data is actually 2.5D, not really 3D information. The photo information also only reflects the percentage of visible green at that moment at a certain point, and the deviation of camera angle will greatly affect the GVI results. Second, the extent to which GVI, as a human visual perception, will affect human emotions deserves further study in the future. Furthermore, in the selection of variables, the nature of land use will also affect the spatial differentiation of GVI, such as the layout of land for cultural facilities, education and science, and administrative offices, etc. However, it is difficult to obtain these data; moreover, we believe that GVI is also closely related to the landscape pattern of each neighborhood, the biological characteristics of forest trees, and the structure of forest stands. Finally, in future work, the dynamic changes of GVI in time can be further studied, which will be more beneficial to determine the key factors affecting the spatial differentiation of GVI in different periods.

## Conclusion

In this study, we used Internet data to crawl the GVI of the main neighborhoods in the central city of Fuzhou and further analyzed the main influencing factors of their spatial differentiation. The results show that GVI in Fuzhou is unevenly distributed and exhibits significant spatial autocorrelation. Areas with high GVI were mainly distributed in the western part of the study area, while areas with low GVI were mainly distributed in the eastern part of the study area; the main factors that caused the spatial divergence of GVI among neighborhoods in Fuzhou were NDVI, house prices, accessibility of water bodies and parks, and the proportion of built space, rather than the proportion of green space. And the range of influence of each variable varies spatially. Therefore, we propose to add new parks and green spaces such as neighborhood parks, waterfront parks, and wetland parks in the southern part of Fuzhou city, and to promote a reasonable increase in urban water bodies in order to enhance the GVI of local neighborhoods. At the same time, we propose to increase rooftop gardens, vertical greening, and green belts on sidewalks in order to improve the NDVI of the city. The results of the study provide some scientific basis for promoting the equity of green vision and maintaining sustainable urban development.

## Data Availability

All datasets generated during and/or analysed during the current study are available from the corresponding author on reasonable request.
